# Overexpression of a Modified Plant Thionin Enhances Disease Resistance to Citrus Canker and Huanglongbing (HLB)

**DOI:** 10.3389/fpls.2016.01078

**Published:** 2016-07-22

**Authors:** Guixia Hao, Ed Stover, Goutam Gupta

**Affiliations:** ^1^U.S. Horticultural Research Laboratory, United States Department of Agriculture, Agricultural Research ServiceFort Pierce, FL, USA; ^2^Los Alamos National LaboratoryLos Alamos, NM, USA

**Keywords:** *Xanthomonas citri*, *Candidatus* Liberibacter asiaticus, modified plant thionin, gene cloning and expression, disease resistance, transgenic plant

## Abstract

Huanglongbing (HLB or citrus greening disease) caused by *Candidatus* Liberibacter asiaticus (Las) is a great threat to the US citrus industry. There are no proven strategies to eliminate HLB disease and no cultivar has been identified with strong HLB resistance. Citrus canker is also an economically important disease associated with a bacterial pathogen (*Xanthomonas citri*). In this study, we characterized endogenous citrus thionins and investigated their expression in different citrus tissues. Since no HLB-resistant citrus cultivars have been identified, we attempted to develop citrus resistant to both HLB and citrus canker through overexpression of a modified plant thionin. To improve effectiveness for disease resistance, we modified and synthesized the sequence encoding a plant thionin and cloned into the binary vector pBinPlus/ARS. The construct was then introduced into *Agrobacterium* strain EHA105 for citrus transformation. Transgenic Carrizo plants expressing the modified plant thionin were generated by *Agrobacterium*-mediated transformation. Successful transformation and transgene gene expression was confirmed by molecular analysis. Transgenic Carrizo plants expressing the modified thionin gene were challenged with *X. citri* 3213 at a range of concentrations, and a significant reduction in canker symptoms and a decrease in bacterial growth were demonstrated compared to nontransgenic plants. Furthermore, the transgenic citrus plants were challenged with HLB via graft inoculation. Our results showed significant Las titer reduction in roots of transgenic Carrizo compared with control plants and reduced scion Las titer 12 months after graft inoculation. These data provide promise for engineering citrus disease resistance against HLB and canker.

## Introduction

Citrus greening (Huanglongbing, HLB) is considered to be the most devastating citrus disease worldwide (Bové, 2006). The disease is mainly associated with *Candidatus* Liberibacter asiaticus (Las) transmitted by the Asian citrus psyllid (ACP), *Diaphorina citri*. The first HLB infected tree was discovered in Florida in 2005 (Brlansky and Rogers, 2007), and HLB has now been found in California and Texas as well. Citrus production in Florida continues to decline, fruit quality is diminished, and production costs have greatly increased. Las is the species associated with HLB-affected trees in North America and its genome sequence has been determined (Duan et al., 2009). HLB pathogenesis is not well understood, due in part to inability to culture the Las bacteria. No effective strategies to eliminate HLB disease and no HLB-resistant citrus cultivars have been identified, though thermal-therapy, chemotherapy, and psyllid management may prolong production in infected trees (Hoffman et al., 2013; Stansly et al., 2014; Zhang et al., 2014). Furthermore, citrus canker disease incited by *Xanthomas citri* imposes additional pressure on the Florida industry threatened by HLB (Gottwald, 2007).

Producing resistant cultivars through conventional breeding is a long-term process due to the long juvenile period in citrus. In addition, Florida citrus production is dominated by a few cultivars with traits not easily reproduced through conventional breeding. Therefore, use of genetic engineering to introduce resistance genes is attractive. Studies have demonstrated that expression of an attacinA gene from *Tricloplusia ni* and a spermidine synthase gene from apple in transgenic citrus conferred considerable canker resistance (Boscariol et al., 2006; Fu et al., 2011). Expression of a dermaseptin gene in sweet orange plants reduced citrus canker symptoms (Furman et al., 2013). In addition, genes associated with plant immunity have been introduced to citrus to enhance citrus canker resistance (Mendes et al., 2010; Zhang et al., 2010; de Oliveira et al., 2013; Hao et al., 2016). Recently transgenic citrus expressing a NPR1 gene from Arabidopsis was reported to enhance HLB resistance (Dutt et al., 2015).

Plants can produce antimicrobial proteins as first line of defense against invading plant pathogens. Antimicrobial proteins include a variety of small peptides such as lipid transfer proteins, plant defensins, and thionins (Kader, 1996; Castro and Fontes, 2005; Pelegrini and Franco, 2005). Thionins are cysteine-rich peptides which show antibacterial, antifungal, anticancer and cytotoxic activities (Guzmán-Rodríguez et al., 2015). Typically a thionin contains an N-terminal signal peptide for targeting the endoplasmic reticulum (ER), a positively charged mature domain with conserved cysteine residues and a C-terminal acidic peptide with no consistent motif. Thionins are usually processed to a mature peptide (44–47 amino acids) that have a characteristic three dimensional structure stabilized by six to eight cysteine residues (Pelegrini and Franco, 2005). Recently it was reported that Arabidopsis thionin Asthi2.4 appeared not to be processed at the C-terminal region (Asano et al., 2013). Different types of thionins are classified on the basis of the net charge, the number of amino acids and the disulfide bonds and their 3-D structure of the mature protein. The α/β -thionins consist of two a-helixes, a double-stranded β-sheets and a C-terminal coil region. The γ-thionins contain one α-helix and three anti-parallel β–sheets which form the typical amphipathic two layer α/β sandwich (Pelegrini and Franco, 2005; Lacerda et al., 2014).

Thionins are postulated to induce the opening of pores on the cell membranes of the pathogen, resulting in leakage of potassium and calcium ions from their cells (Pelegrini and Franco, 2005; Oard, 2011). It has been demonstrated that sub-inhibitory concentrations of a hordothionin in barley increased Ca^2+^ uptake in hyphae of *Neurospora crassa* (Thevissen et al., 1996). In addition, α-hordothionin also caused increased K^+^ efflux and alkalization of the medium, leading to rupture of the membrane lipid bilayers (Oard, 2011). Thionin protein accumulation was reported to be increased in wheat cell walls after inoculation with *Fusarium culmorum*, which suggested that cell wall accumulation of thionins in infected wheat spikes may be associated with defense responses against *F. culmorum* or *F. graminearum* (Kang and Buchenauer, 2003). Recently, the secreted antifungal thionin Asthi2.4 has been shown to suppress the toxicity of a fungal fruit body lectin from *F. graminearum* (Asano et al., 2013).

The objective of our research is to transform commercial varieties of citrus with genes for HLB and canker resistance. In an earlier report, we showed that transgenic tobacco expressing a modified thionin remarkably enhanced disease resistance incited by *Pseudomonas tabaci* (Hao et al., unpublished). In this study, we characterized and evaluated the expression of endogenous citrus thionin homologs. Since endogenous citrus thionin expression is not sufficient to protect against HLB and canker disease, we designed, synthesized, cloned and introduced a modified thionin into Carrizo, generating transgenic plants. We confirmed gene integration and expression in the transgenic plants by PCR and RT-qPCR. We showed that overexpression of thionin significantly increased canker resistance and inhibited canker bacterial growth in the transgenic Carrizo. We also demonstrated a significant reduction in Las titer in transgenic Carrizo roots following grafting with Las-infected rough lemon scions, and after 12 months Las titer was also much lower in scions on the transgenic Carrizo.

## Materials and methods

### Citrus thionin characterization, comparison, and phylogenic analysis

Basic local alignment search tools (BLAST) were used to search thionin homologs in NCBI and the citrus genome database (http://www.phytozome.net). Sequence alignment was performed with NTI vector and MEGA program. A phylogenic tree was constructed using the MEGA 6 program (Tamura et al., 2011) with the Maximum likelihood method (Whelan and Goldman, 2001) using a bootstrap value of 1000.

### Molecular analysis of citrus thionin expression

RNA was extracted from leaves, stems and roots of 2-month old Hamlin and Carrizo using Trizol reagent according to the manufacturer's instructions (Sigma-Aldrich, St. Louis, MO). Total RNA was quantified using the Nanodrop and treated with RQ1 RNase-free DNase from Promega Corp (Madison, WI). DNase-treated RNA (~1.5 μg) was used to synthesize first-strand cDNA with 0.5 μg of oligo (dT) primer and 1 μL of SuperScript® III reverse transcriptase in a 20 μL reaction (Invitrogen). A negative control without the reverse transcriptase was performed to verify absence of genomic DNA contamination. RT-qPCR was run and analyzed on a real time PCR machine ABI7500 using cDNA as described (Hao et al., 2016). Primers for *CsthiF, Csthi1*, and *Csthi2* are listed in Table 1. The citrus gene Glyceraldehyde-3-phosphate dehydrogenase C2 (*GAPC2*) was amplified with primers Cs*GAPC2-5*′ and Cs*GAPC2-*3′ and used to normalize the values as an internal control (Table 1). The gene expression levels in leaves, stems and roots of Hamlin and Carrizo were compared by 2^ΔCt^ fold which was obtained for each sample versus a reference sample which had the lowest 2^Δ*Ct*^ in the tested samples. ΔCt = Ct (internal control) - Ct (target gene). The qPCR reactions were set up in triplicate and repeated twice with similar results.

**Table 1:** **Primers used in this study**.

**Gene name**	**Primer sequence**
**FOR TRANSGENIC PLANTS**	
D35S-For	5′-GACGCACAATCCCACTATCC-3′
Nos-Rev	5′-TTTGCGCGCTATATTTTGTTT-3′
**FOR GENE EXPRESSION BY REAL TIME PCR**	
*CsthiF*-RT-5′	5′-AGAACACATAGCAGAGCTTTCA-3′
*CsthiF*-RT-3′	5′-GAGTGCTGTACAGTAGCCATAG-3′
*Csthi1*-RT-5′	5′-GGGCCATGTGTGAGTAAGAG-3′
*Csthi1*-RT-3′	5′-AAAGCATCGACGACGGAAT-3′
*Csthi2*-RT-5′	5′-GGCTAAGTCAGTTGCTAGCATTA-3′
*Csthi2*-RT-3′	5′-CCGAAGGAAGCAAAGAGTATGA-3′
*Mthionin*-RT-5′	5′-TTTCGCCGTAGATGCAGATG-3′
*Mthionin*-RT-Nos-3′	5′-TCCTAGTTTGCGCGCTATATTT-3′
*CsGAPC2*-RT-5′	5′-TCTTGCCTGCTTTGAATGGA-3′
*CsGAPC2*-RT-3′	5′-TGTGAGGTCAACCACTGCGACAT-3′
**FOR *X. CITRI* GROWTH DETECTION**	
VM3	5′-GCATTTGATGACGCCATGAC-3′
VM4	5′-TCCCTGATGCCTGGAGGATA-3′
**FOR LAS DETECTION**	
LL-F	5′-CTTACCAGCCCTTGACATGTATAGGA-3′
LL-R	5′-TCCCTATAAAGTACCCAACACTAGGTAAA-3′
CD-F	5′-TGAGTACGAGCCGAGTGTTG-3′
CD-R	5′-CTGGTGGATCGGTGAAGTTT-3′

### Vector construction

A synthetic gene encoding the modified thionin (Mthionin) was generated by DNA2.0 (Menlo Park, CA). The synthesized genes were isolated by digestion of the vector with Sma I and Kpn I and were ligated to binary vector pBinPlus/ARS, which contains double 35S (D35S) promoter to drive Mthionin expression and the kanamycin resistance gene (nptII) to facilitate selection of plant transformants. The resulting plasmid was used to transform *E. coli* TOPO10 competent cells (Invitrogen, CA). The positive clone was picked up by colony PCR, followed by plasmid isolation and sequencing to confirm the consensus clone. The binary vector containing the peptide gene was introduced into *A. tumefaciens* EHA105 by electroporation for plant transformation.

### Transformation and generation of transgenic citrus

Transformation of citrus was performed using *Agrobacterium*-mediated transformation (Hao et al., 2016). Citrus epicotyls were transformed with the *Agrobacterium* strain EHA105 carrying the *Mthionin* vector. To test if the T-DNA region was integrated into the citrus genome, primers D35S-F and Nos-R were designed to span from with D35S promoter to the Nos terminator region (Figure S1). Citrus genomic DNA was isolated with the Qiagen plant kit (Qiagen, Valencia, CA). The D35S promoter primer and the Nos terminator primer were used to amplify plant genomic DNA using Taq polymerase (Table 2). The PCR reactions were performed and the PCR products were run on 1% agarose gel and stained with ethidium bromide.

**Table 2:** **Disease development at 10 days following infiltration with *X. citri* 3213 in transgenic Carrizo expressing Mthionin**.

**Transgenic Plant**	**Bacterial Infiltration Level (CFU/ml)**			
	**10^4^**	**10^5^**	**10^6^**	**10^7^**
Neg. Control 1	++	+++	+++++	+++++
Neg. Control 2	++	+++	+++++	+++++
MThionin-C1	+∕−	++	+++	++++
MThionin-C3	−	−	+∕−	++++
MThionin-C5	+∕−	+	+++	++++
MThionin-C12	−	−	+	+++
MThionin-C13	−	−	+∕−	+++
MThionin-C14	−	−	+∕−	+++
MThionin-C15	−	−	++	+++
MThionin-C16	−	−	+	+++
MThionin-G	−	−	+∕−	+++
MThionin-H	−	−	++∕−	+++
MThionin-I	−	−	+∕−	+++

*−indicates no canker observed*.

*+indicates canker observed*.

*(The number of “+” indicates severity of disease observed with “+++++” being highest)*.

### RNA isolation and reverse transcriptase real time PCR (RT-qPCR)

RNA extraction, cDNA amplification and RT-qPCR were performed as described above. Primers for the modified thionin sequence were designed as Mthionin-5′ located in modified gene and Mthionin-3′ located in Nos region (Table 1 and Figure S1). The citrus gene Cs*GAPC2* was used to normalize the values as an internal control. Gene expression level was compare with the 2^Δ*Ct*^ values as described above. The qPCR reactions were set up in triplicate and repeated twice with similar results.

### Canker pathogenicity assays

To challenge transgenic Carrizo lines, overnight cultures of *X. citri* strain 3213 were centrifuged and diluted to OD_600_ of 0.3 with sterile distilled water and further diluted to 10^4^, 10^5^, 10^6^, and 10^7^ CFU/ml (Hao et al., 2016). The bacterial suspensions were infiltrated from the abaxial side into leaves of transgenic citrus plants containing our constructs and controls using a syringe. Inoculated plants were incubated for 10 days and then the disease development was scored and photographed. Spray inoculation was performed with a 10^6^ CFU/mL suspension of *X. citri* containing 0.02% silwet-77. Three young fully expanded leaves were spray-inoculated on transgenic and control plants. Disease severity was evaluated frequently after inoculation. Images were taken 4 weeks after spray inoculation.

To investigate bacterial growth rate in transgenic citrus plants, *X. citri* (10^6^ CFU/ml) was infiltrated into leaves of control and transgenic citrus lines. Leaf disks (0.5 cm diameter) were punched from the infiltrated area with a cork borer at 2 h, 3, 6, 9, and 12 d after inoculation. DNA was isolated from leaf disks and bacterial populations at each time point were measured by qPCR as described (Stover and McCollum, 2011).

### HLB challenge via grafting transmission and Las detection by qPCR

Transgenic Carrizo expressing Mthionin or vector alone were propagated by rooting cuttings alongside nontransgenic controls. Transgenic plants tested were selected based on levels of Mthionin expression and canker evaluation results. The following experiment was used for HLB challenge via graft transmission: three nontransformed Carrizo (CK); six replicated transgenic plants expressing vector only (CKT1 and CKT2); replicated transgenic Carrizo lines Mthionin-C14 (seven plants), C26 (three plants), G (six plants), H (five plants), and I (four plants). The budwood used for inoculation was rough lemon infected with Las. Each plant was grafted with two HLB-infected rough lemon buds, which were shown to have high Las titer by qPCR (Ct values ranging from 24.0 to 26.0). Grafted plants were grown in the greenhouse. Symptoms were evaluated periodically. Fibrous roots were sampled at 9 months post graft inoculation. To more comprehensively examine HLB development in transgenic and control plants, old leaves (most with HLB symptom), young expanded leaves (with or without HLB symptom) and fibrous roots were sampled at 12 months post graft inoculation. DNA from leaf midribs was isolated with the Qiagen plant kit. Root DNA was isolated using the PowerSoil DNA Isolation Kit (Mo Bio Laboratories) as described (Johnson et al., 2014). Las titer was tested by qPCR with Bright Green PCR Master Mix (Sigma-Aldrich, St. Louis, MO) using an ABI7500 thermal cycler (Thermo Fisher Scientific, Waltham, MA) as described. Las long primers were used for Las detection and citrus dehydrogenase (CD) was amplified and used to examine DNA quality (Table 1). The qPCR was performed in triplicate and repeated twice.

## Results

### Characterization of endogenous citrus thionin homologs

**Figure 1:**
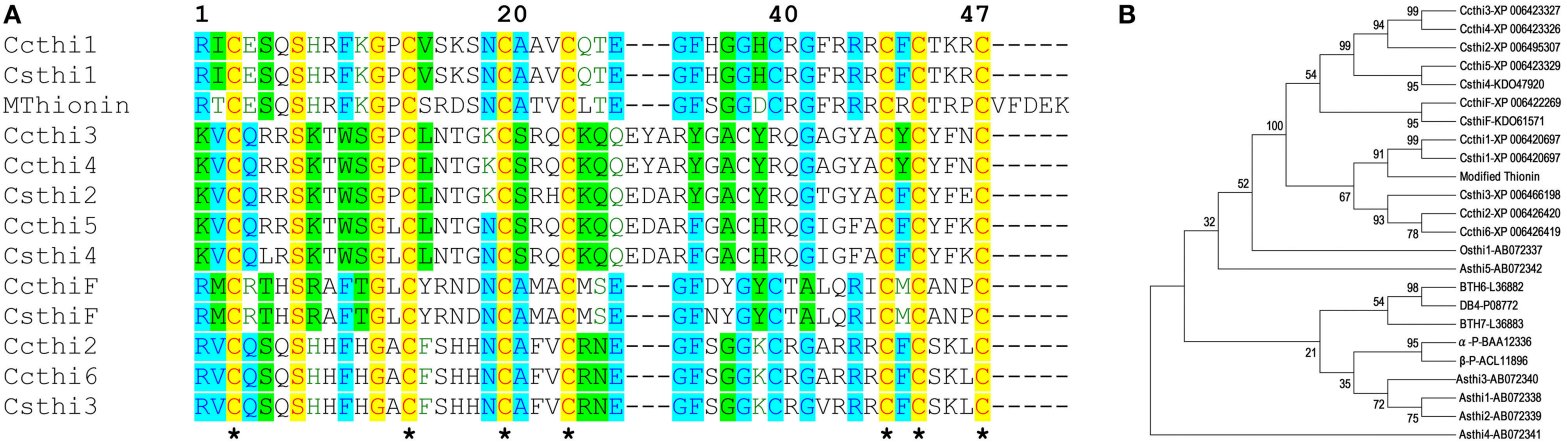
**(A)** Alignment of the modified thionin amino acids sequence with endogenous citrus thionin homologs by NTI alignment program (Invitrogen). Conserved cysteine residues are shaded in yellow and marked with ^*^. **(B)** Phylogenetic tree to show relationship among modified thionin, citrus, barley, wheat, oats, and rice thionin homologs. Citrus thionin homologs were retrieved from BLAST of citrus database and NCBI database. MEGA 6 program was used to construct the tree using Maximum likelihood method with 1000 bootstraps. The sequences of *Asthi1* through *Asthi5* are available as GenBank accession numbers AB072338 to AB072342, and *Osthi1* is GenBank accession number AB072337. The thionin homologs from barley BTH6 (GenBank accession number L36882), BTH7 (GenBank accession number L36883), DB4 (GenBank accession number P08772), and from wheat α-and β-purothionin (GenBank accession number BAA12336 and ACL11896) are also included in the phylogenetic analysis.

In the *C. sinensis* genome (www.phytozome.net and NCBI), we identified one thionin homolog designed as CsthiF (KDO61571). CsthiF contains 555 nucleotides (nt) encoding 184 amino acids (aa). In the *C. clementina* genome, a similar thionin homolog encoding 184 aa was designated CcthiF (Xp_006422269). CsthiF and CcthiF proteins share 100% identity and both contain three conserved domains characteristic of a plant thionin family: a signal peptide, a mature thionin domain and an acidic protein domain. The mature forms of the CsthiF and CcthiF peptides contain 47 amino acids with eight conserved cysteine (Cys) residues (Figure 1A). A 100-bp intron was identified between the signal peptide and mature domain.

In addition to full length CsthiF and CcthiF genes, several thionin homologs were identified in citrus genomes, which contained a signal peptide and a mature thionin domain but lacked an acidic protein domain. In *C. sinensis*, four thionin homologs were identified. Csthi1 (orange 1.1g034999 m, identified in www.phytozome.net) encodes 76 aa including a 29-aa signal peptide and 46-aa mature domain, which has no homolog present in *C. sinensis* genome from NCBI data but shares high identity (100%) to Ccthi1 (XP_006420697) in *C. clementina*. Csthi2 (XP_006495307) encodes 82 aa with a 28-aa signal peptide and a 54-aa mature form. Csthi3 (XP_006466198) encodes 73 aa with a 26-aa signal peptide and a 47-aa mature form. Csthi4 (KDO47920) encodes 78 aa with a 28-aa signal peptide and a 50-aa mature form. Csthi1 shares 21% identity to Csthi2, 51% to Csthi3, and 26% to Csthi4 at the protein level. Six thionin homologs were identified in the *C. clementina* genome and these were designed as Ccthi1 (XP_006420697), Ccthi2 (XP_006426420), Ccthi3 (XP_006423327), Ccthi4 (XP_006423326) Ccthi5 (XP_006423329), and Ccthi6 (XP_006426419). There is some variation between these homologs in the intron and untranslated region, however Ccthi1 showed 100% homology with Csthi1 and Ccthi2 showed 55% identity to Ccthi1. Ccthi3, Ccthi4, and Csthi2 share 100% homology. Ccthi5 shares 99% identity to Csthi4 and Ccthi6. All these mature thionins contain eight conserved cysteine residues (Figure 1). Phylogenetic analysis was performed using the MEGA 6 program (Tamura et al., 2011) to determine similarity among these citrus thionins and other thionin homologs from oats, barley, wheat and rice. Our results showed citrus thionins share high similarity with thionin homologs from oats (Asthi5) and rice (Osthi1; Figure 1B).

### Native thionin expression varied in different citrus tissues

**Figure 2:**
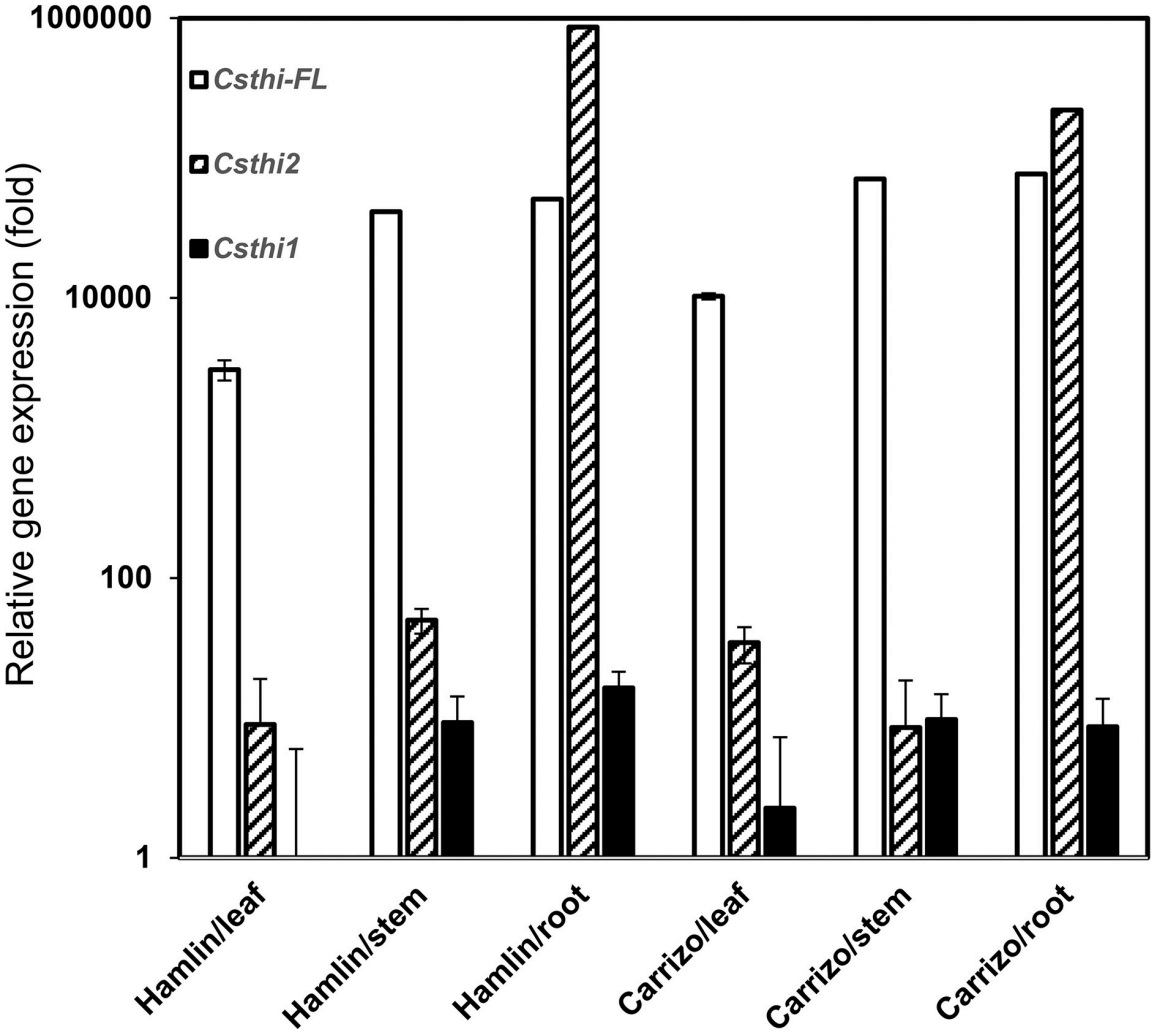
**Endogenous citrus *thionin* expression from different organs of Hamlin and Carrizo**. The samples were normalized against citrus Glyceraldehyde-3-phosphate dehydrogenase C2 (*CsGAPC2*) gene. Expression fold change is relative to the lowest observed thionin expression which was *Csthi1* in Hamlin leaves. The experiments were set up as triplicate and repeated twice.

Thionin expression has been reported in seeds, stems, roots, leaves, and flowers in many plant species (Silverstein et al., 2005). To further characterize citrus thionin homologs, three sets of primers were designed based on sequence alignment and phylogenetic analysis. We examined the expression of *CsthiF* (the only full length thionin identified in citrus), *Csthi1* and *Csthi2* in healthy leaves, stems and roots of 2-month-old Hamlin sweet orange and Carrizo citrange by RT-qPCR. *Csthi1* had the lowest expression in Hamlin leaves, which was therefore used as a reference (designated as having expression = 1) in analysis of thionin expression. Compared to leaves, relatively high expression of *Csthi1* was observed in stems and roots of Hamlin and Carrizo (about 10 to 20-fold higher than in Hamlin leaves; Figure 2). The expression of *CsthiF* was observed to be thousands-fold higher than Csthi1 expression, but with generally less expression in leaves, and 15 to 25-fold higher levels in stem and roots of Hamlin and Carrizo (Figure 2). Interestingly, the RNA abundance of *Csthi2* in Hamlin and Carrizo roots was over 50,000 times higher than the expression of *Csthi1* and *CsthiF*. In contrast, only very low levels of expression of *Csthi2* were observed in leaves and stems of Hamlin and Carrizo (Figure 2).

### Generation of transgenic citrus overexpressing mthionin

Thionins are folded plant peptides with anti-fungal and antibacterial activity. Though there are high levels of endogenous thionin expression (*CsthiF* and *Csthi2*) in *Citrus*, no HLB resistant cultivar has been identified within the genus. Carrizo is reported to be somewhat resistant to HLB when grown as an ungrafted tree but is susceptible to canker disease. Hamlin sweet orange is susceptible to HLB and canker. These suggest that the expression of endogenous thionin is not enough to prevent HLB and canker disease development. Among plant thionins, gamma-thionins are particularly effective against gram-negative bacteria (Lacerda et al., 2014). Critical to their function are: 4 conserved Cys-Cys bridges and residue arginine between 6th and 7th Cysteine. In addition, other basic residues arginine and lysine in the thionin may also play a role in bacterial membrane lysis. Therefore, it is critical to examine the energetically stable tertiary folds that retain the conserved Cys-Cys bridges and optimally exposes basic residues to interact with the bacterial membrane in order to limit toxicity. To improve thionin contribution to disease resistance, a modified thionin (Mthoinin) was designed to enhance its activity by optimally exposing leucine and arginine, optimizing stable tertiary fold with beta sheet and helix as well as the unspecified residues in coil structure. Five extra amino acids were added at the C terminus to create Mthoinin because structure in the C-terminal acidic region can increase the stability and solubility of the protein in many thionins (Romero et al., 1997). Our Mthionin contains both hydrophobic and hydrophilic domains, which can target the outer-membrane proteins and lipid A of the lipopolysaccharide (LPS) of gram-negative bacteria. The hydrophobic domain binds to the hydrophobic lipid A, whereas the positively charged hydrophilic domain inserts among the negatively charged head groups of LPS. In addition, Mthionin was proven to be effective against *P. syringae* pv. *tabaci* in transgenic tobacco (Hao et al., unpublished). The Mthionin has considerable identity to Csthi1 (64%) which has the lowest expression in Hamlin and Carrizo (Figures 1A, 2).

**Figure 3:**
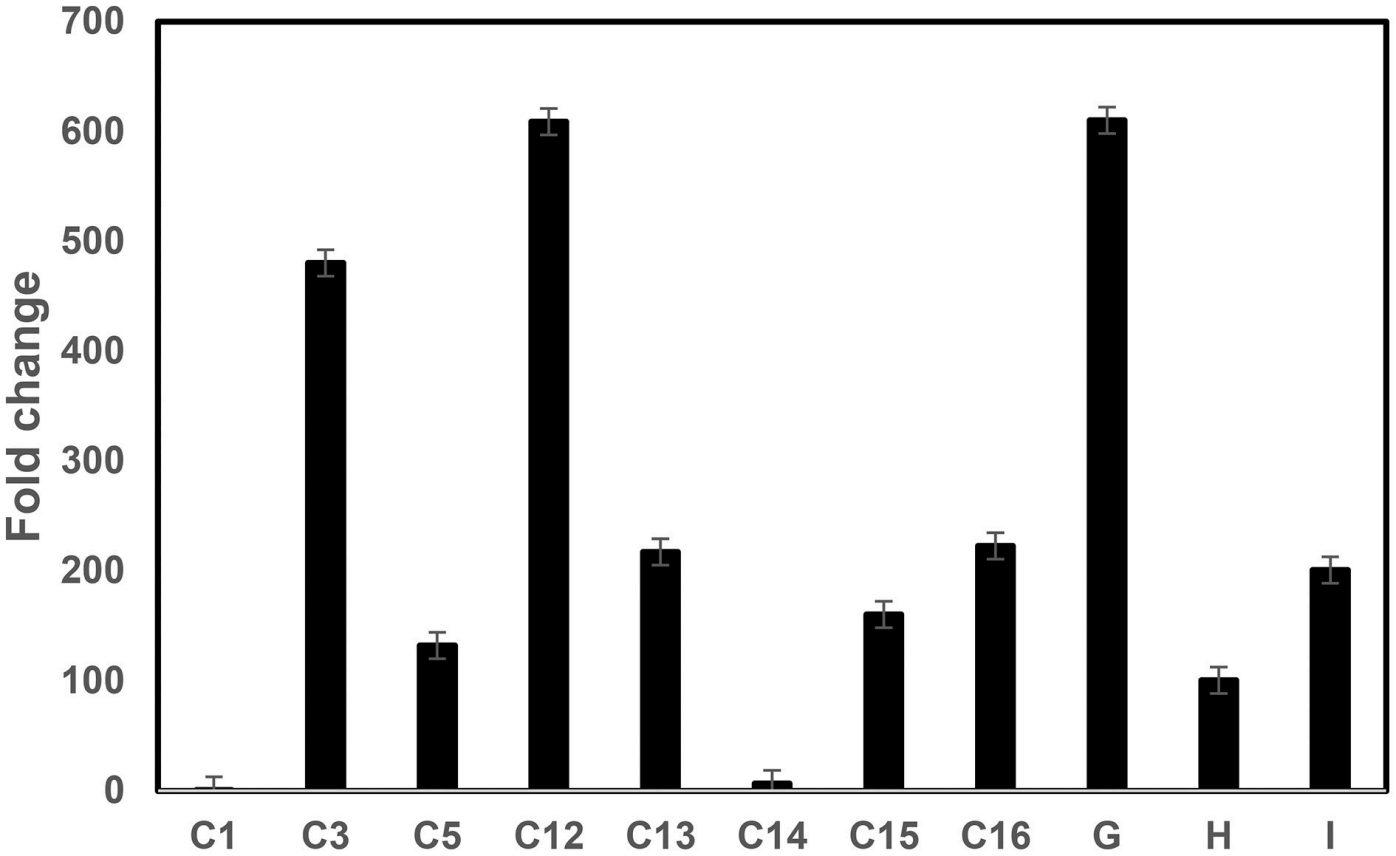
**RT-qPCR comparison of Mthionin transgene expression levels in transgenic Carrizo lines expressing modified *thionin***. Relative gene expression of the target gene was normalized to the expression of the citrus Glyceraldehyde-3-phosphate dehydrogenase C2 (*CsGAPC2*). A ratio of relative gene expression is calculated from the 2^ΔC^ values of a sample vs. Thionin C1 which has lowest 2^ΔC^ in the tested samples. The experiments were set up as triplicate and repeated twice.

The Mthionin gene was synthesized and cloned into the binary vector pBinARS/plus for citrus transformation (Figure S1). Primers were designed to the D35S promoter region and Nos terminal region to verify integration of the Mthionin gene (Table 1). A total 30 putative transgenic Carrizo plants were obtained and PCR detection showed 25 of them carrying the Mthionin gene. The PCR results of 15 independent transgenic plants carrying Mthionin are shown in Figure S2, along with positive control and negative control samples. Transgene expression was determined by real time reverse transcriptase PCR (RT-qPCR; Figure 3). The relative RNA abundance shown in Figure 3, indicates that transgenic plants of thionin C12 and G have higher expression levels compared to the other transgenic lines. All transgenic plants appeared to be normal in growth and phenotype.

### Increased canker resistance in transgenic Carrizo expressing mthionin

**Figure 4:**
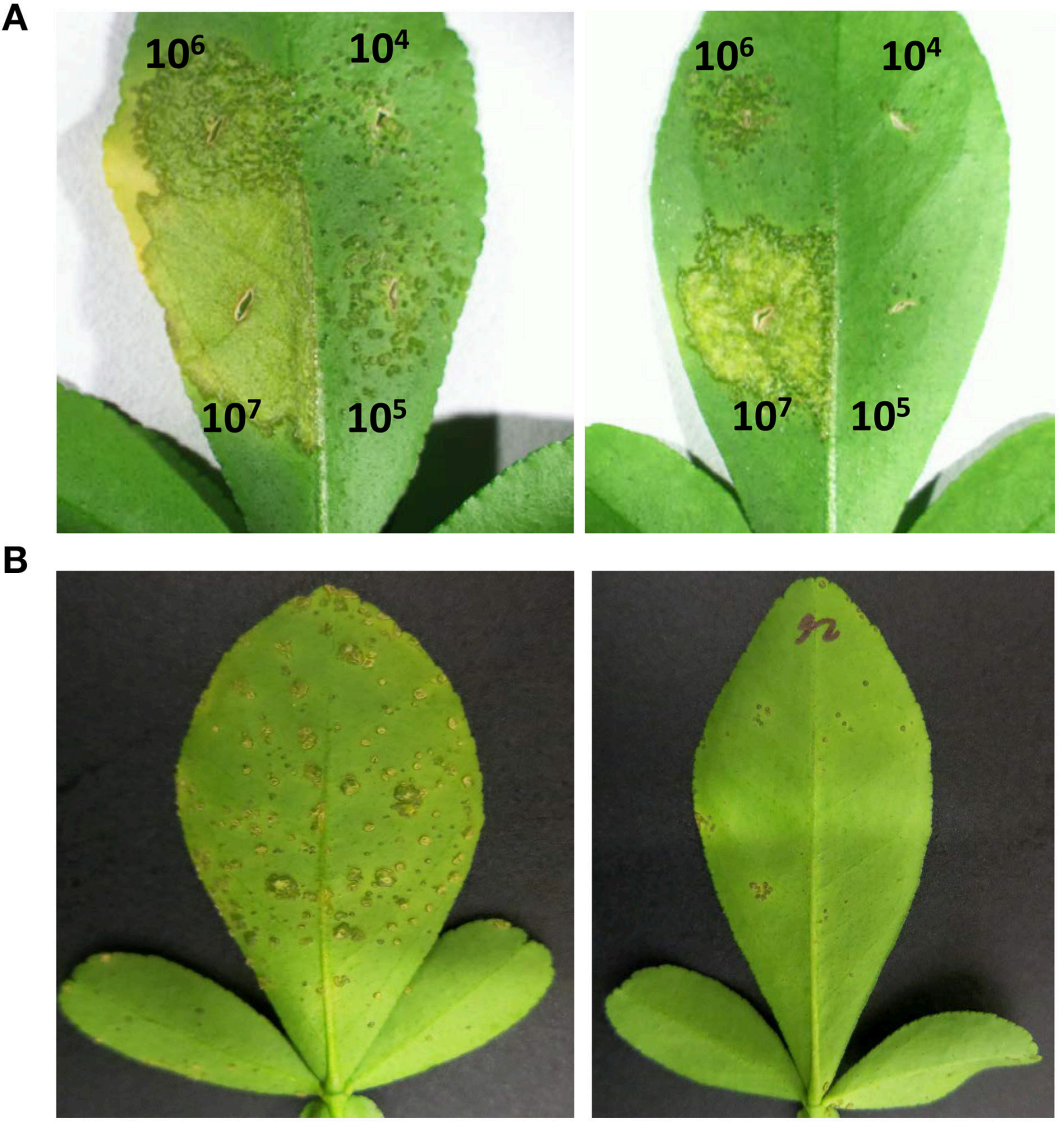
**Canker disease symptoms caused by *Xanthomonas citri* strain 3213 on leaves of control plant (left) and transgenic line C13 expressing thionin (right) by infiltration and spray-inoculation**. **(A)** Infiltration; **(B)** Spray-inoculation. The concentrations of infiltration were adjusted to 10^4^, 10^5^, 10^6^, and 10^7^ CFU/ml. The photograph was taken 10 days after infiltration. The concentration of 10^6^ CFU/ml was used for spray inoculation. The photograph was taken 3 weeks after inoculation.

We evaluated canker development in control plants and transgenic plants expressing Mthionin. Leaf infiltration assays were used to assess canker development incited by *X. citri* strain 3213. Three newly expanded leaves were selected for infiltration with concentrations of 10^4^, 10^5^, 10^6^, and 10^7^ CFU/ml. Canker development was assessed at 10 days after inoculation. Compared to control plants which developed typical canker lesions, most of the transgenic plants expressing Mthionin did not show canker development at 10^4^–10^5^ CFU/ml, and showed reduced canker development at concentrations of 10^6^ CFU/ml and even at 10^7^ CFU/ml (Table 2). Infiltrated zones of transgenic line C13 showed significant canker resistance at all bacterial concentrations compared to control plants (Figure 4A). Furthermore, with spray inoculation at 10^6^ CFU/ml, transgenic plants expressing Mthionin showed marked resistance compare to control plants (Figure 4B).

**Figure 5:**
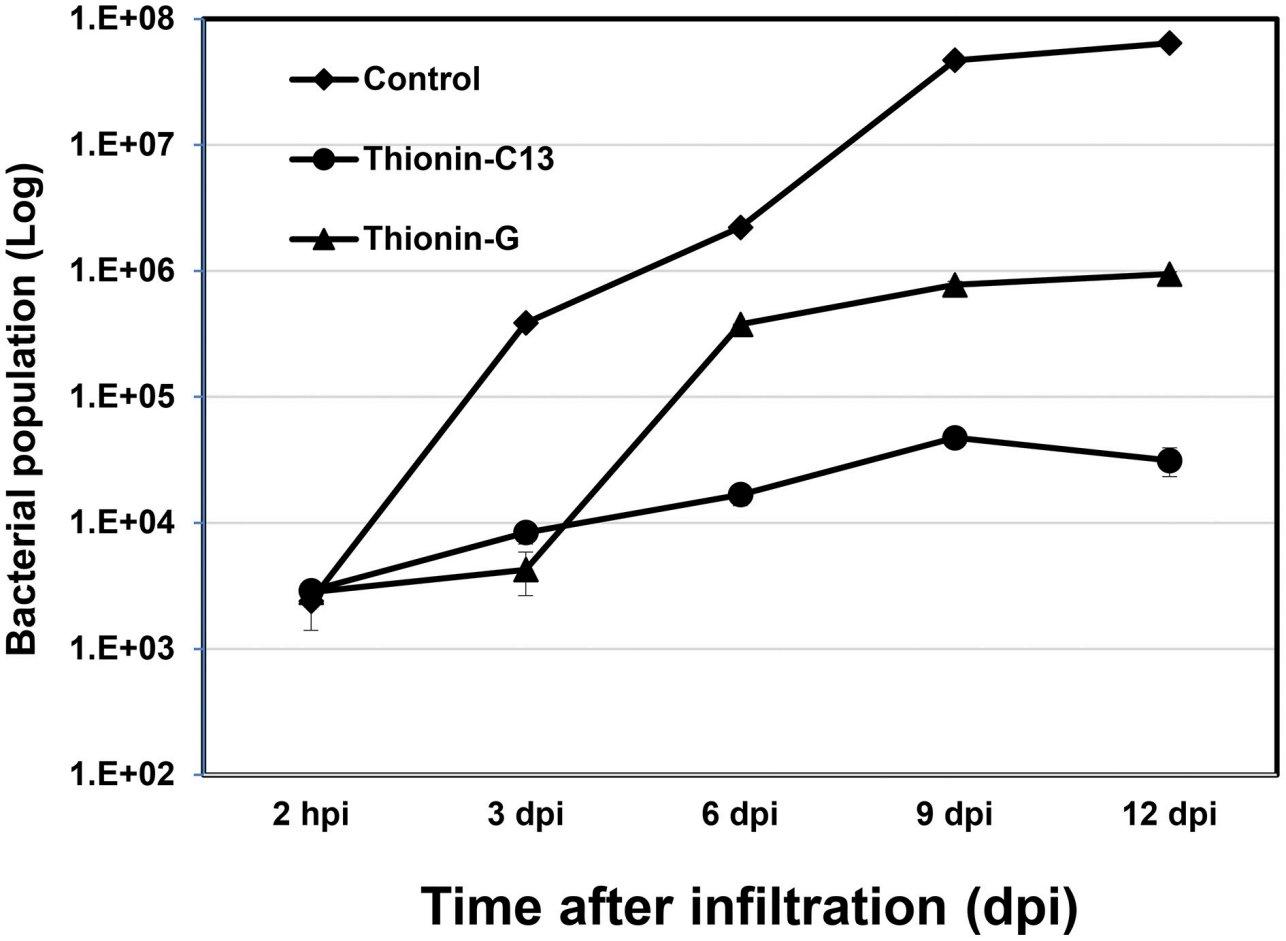
**Bacterial growth in leaves of control and transgenic Carrizo expressing thionin**. Three newly expanded leaves of each plant were infiltrated with ~10^6^ CFU/ml and three leaf disks were taken for DNA isolation from each leaf at 2 h, 3, 6, 9, 12 d post-infiltration. Real time PCR based on *X. citri pthA gene* was used to calculate bacterial population using formula: Copy Number _(*X_cc_, pthA*)_ = 10 ^(38.3−ct)∕3.56^.

*X. citri* growth was also evaluated at various time points after leaf infiltration at 10^6^ CFU/ml in transgenic plants and nontransgenic controls. Much less *X. citri* development was observed in transgenics expressing Mthionin compared to nontransgenic plants (Figure 5).

### Reduced Las titer in transgenic Carrizo expressing mthionin

**Figure 6:**
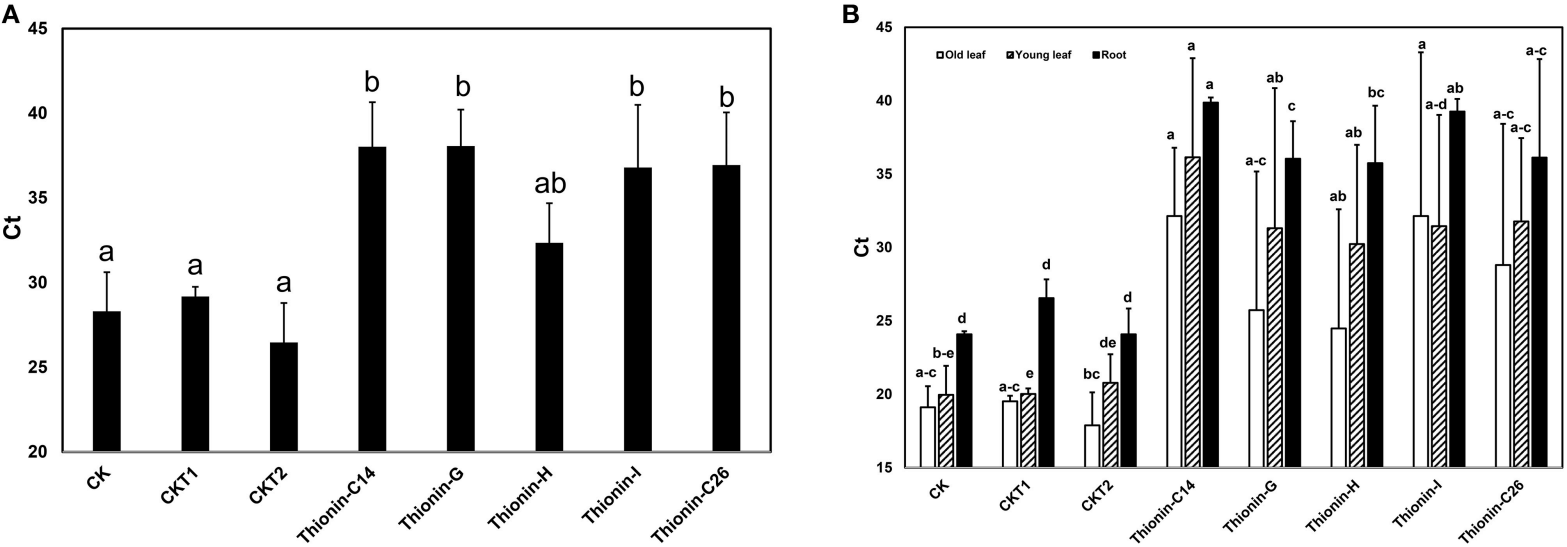
**(A)** Quantification of Las titer from roots of the transgenic plants expressing Mthionin and controls 9 months after grafting with highly infected rough lemon scions. **(B)** Quantification of Las titer from old leaves, new leaves and roots of the transgenic plants expressing Mthionin and controls 12 months after grafting with highly infected rough lemon scions. CK, Nontransformed wild type; CKT1 and CKT2, Transgenic plants expressing vector; Thionin transgenic plants, C14, G, H, I, and C26. The qPCR reactions were set up in triplicate and repeated twice with similar results. Bars represent Ct values obtained by qPCR so lower values indicate higher Las titer. Results are the means of three to seven replicated plants. Statistical analysis was conducted using Kruskal–Wallis at a value *p* < 0.05. Bars marked with the same letters are not significantly different at the *p* < 0.05 level.

Infected rough lemon shoots were used as sources for transmitting Las to the Carrizo rootstock, with the infected rough lemon then maintained as scions. Before grafting, Las titer was tested in infected rough lemon leaves and showed Ct values from 24.6 to 26.2 by qPCR. Wild-type Carrizo and transgenic Carrizo expressing the empty vector were used as controls. Six month after graft inoculation, we detected Las presence in a majority of rough lemon leaves (data not shown). Typical HLB symptoms including blotchy mottle and leaf chlorosis were observed in the majority of rough lemon scions 9 month after grafting. To test whether Mthionin can inhibit Las growth in transgenic rootstocks, DNA was isolated from transgenic roots and control plants at 9 month after grafting. Las titer was obtained with Las specific primers (lower Ct indicates higher bacterial titer). Ct values from individual control and transgenic plants were compared (Table S1). Roots of three transgenic Carrizo plants showed no Las, which may result from failed graft transmission or slow Las growth affected by Mthionin expression. Therefore, Las titer was compared between controls and transgenic plants considering only the trees with detectable Las (Figure 6A): wild-type control and transgenic control plants showed high Las titer with Ct values range from 26.5 to 29.2. Transgenic plants expressing modified thionin showed significantly lower titer with Ct values from 35.4 to 37.5. No obvious rough lemon growth difference was observed between transgenic and control plants (data not shown).

Twelve months after graft inoculation, Las titer was examined and compared from old leaves (most with HLB symptom), young expanded leaves (with or without HLB symptom), and fibrous roots of transgenic and control plants (Table S2, Figure 6B). All plants except thionin-H3 showed Las titer which indicated only one plant with failed graft inoculation from a total of 34 plants. Las titer varied in transgenic plants expressing Mthionin and control plants. In old leaves, Las titer varied from 17.9 to 38.8. In young leaves, no Las was detected in grafted rough lemon on transgenic plants Mthionin C14-1, 2, or 3 and G1, G6, or H5; in contrast high titer was observed in grafted control plants. Some grafted young rough lemon leaves on transgenic root stocks also showed high titer such as in Mthionin C14-6, G2, G3, G4, and I3. It is worth noting that significantly lower Las titer was none-the-less observed in the transgenic root stock for Mthionin C14-6, G2, G3, and I3 though both old and new leaves had high Las titer (Table S2). Statistical analysis of replicates for each Mthionin line and controls showed no difference between wild-type controls and Mthionin plants in older leaves, only Mthionin C14 had lower Las in young leaves, and all Mthionin lines had lower Las titer in roots (Figure 6B). An analysis comparing all Mthionin plants vs all controls showed significantly lower Las titer in Mthionin transgenics in each tested tissue at a *p* < 0.008, with Ct differences indicating an average of about a thousand fold reduction in Las. Taken together, our results indicate that transgenic citrus expressing Mthionin has lower Las titer and is promising for HLB resistance. HLB develops slowly and more time and tests are needed to confirm transgenic plant disease resistance in the greenhouse and field.

## Discussion

In this study, we characterized endogenous citrus thionin homologs and compared their expression in different citrus tissues. We synthesized, cloned and expressed a modified thionin (Mthionin) in transgenic citrus. We showed that transgenic citrus overexpressing the Mthionin had enhanced disease resistance to citrus canker and greatly reduced *X. citri* growth. Further we determined that transgenic Carrizo plants expressing the Mthionin had significantly reduced Las titer in roots 9 months after graft inoculation and when all Mthionin plants were contrasted with all controls at 12 months, Mthionin transgenics averaged about a thousand fold reduction in Las in old and new leaves of the non-transgenic rough lemon scions as well as in roots. Our results suggest that overexpression of a plant thionin can enhance resistance to citrus canker and HLB and perhaps other citrus diseases.

Thionins which show antimicrobial activity against plant pathogens have been identified in many plants such as barley and soybean (Bohlmann et al., 1988; Choi et al., 2008). One full length thionin was identified from citrus which contains a signal peptide, a mature domain and an acid domain, which has relatively high expression in stem and root of Hamlin and Carrizo (Figure 2). Surprisingly, several thionin homologs were identified in citrus without the typical C-terminal domain. The function of the C-terminal peptide is not clear, but it has been proposed to balance the cationic primary thionin domain. Typically thionins are processed to 5 kD mature peptides, however it was discovered that thionin Thi2.4 from Arabidopsis is not processed and this may be important for its activity against the fugal pathogen *Fusarium graminearum* (Asano et al., 2013).

Transgenic plants overproducing thionins have shown resistance to several bacterial diseases in rice and potato (Iwai et al., 2002; Muramoto et al., 2012). Transgenic Arabidopsis and tomato plants overexpressing Arabidopsis thionin 2.1 (Thi2.1) show enhanced resistance to multiple bacterial and fungus diseases (Epple et al., 1997; Chan et al., 2005). Studies showed pyrularia thionin from the nuts of *Pyrularia pubera* and viscotoxins from *Viscum album* are hemolytic and cytotoxic (Vernon, 1992). However, thionins from grains such as wheat have no toxic activity (Vernon, 1992). To facilitate the correct folding and reduce toxicity, the synthetic thionin peptide in our construct was modified by amino acid substitution and addition of extra amino acids at C-terminus. Compared to native citrus thionin, about 30% of the amino acids were substituted and five extra amino acids added at C-terminal of the peptide. It has been demonstrated that the C-terminal acidic stretch can increase the stability and solubility of the protein in many thionins (Romero et al., 1997). In addition, it has been reported that Arg substituted for Phe at the C-terminus reduced native thionin toxicity (Pelegrini and Franco, 2005). Since Las is phloem-limited bacteria which cause HLB, phloem specific expression promotor will be used to drive Mthionin expression in transgenic plants which will reduce potential toxicity in citrus fruits and produce consumer-friendly transgenic plants.

We characterized endogenous citrus thionin homologs and determined their expression in different citrus tissues. Native expression levels are clearly not sufficient to suppress canker and HLB disease development. However, many studies report reduced HLB symptoms and Las titers in *Poncirus trifoliata* and its hybrids with *Citrus*, such as Carrizo (*Citrus sinensis* × *P. trifoliata*) which suggests that these genotypes are tolerant to HLB (Folimonova et al., 2009; Albrecht and Bowman, 2012). It will be interesting to determine whether expression of these thionins can be induced by exposure to pathogens such as Las and *X. citri*.

RT-qPCR showed varying levels of Mthionin expression in the different transgenic plants. Gene expression was higher in the transgenic plants thionin C12 and G compared to transgenic lines C1 and C14 (Figure 3). No Mthionin expression was detected in transgenic plant C1, despite presence of the transgene. Gene silencing can be induced by homologs when foreign genes are highly expressed (Rajeevkumar et al., 2015). Though the thionin gene was modified in our constructs, it still shares about 60% homology with Csthi1. Some degree of correlation has been observed between the level of transgene expression and disease resistance (Lu et al., 2013), but it is not always the case in our studies. Our results showed that high transgene expression in line Mthionin-G provided disease resistance. In contrast, low gene expression was detected in transgenic line Mthionin C14, and significant canker and HLB resistance were still observed. A good correlation was demonstrated between the levels of lactoferrin protein produced in transgenic wheat lines and the level of resistance against *F. graminearum* (Han et al., 2012). It is possible that Mthionin peptide levels are correlated with canker and HLB resistance. Further investigations are underway to produce Mthionin monoclonal antibody and use Western blots to quantify levels of Mthionin peptide.

It has been demonstrated that overexpression of a thionin protein in transgenic rice resulted in its accumulation in cell walls and stopped the invasion of *Burkholderia plantarii* at the surface of stomata (Iwai et al., 2002). In our infiltration assay, when the inoculation concentrations were at 10^6−7^ CFU/ml, less extensive canker lesions developed and were restricted to the infiltrated zones in transgenic plants expressing Mthionin. When the infiltration concentrations were reduced to 10^4−5^ CFU/ml, very few lesions were observed in the transgenic plants in contrast to controls. In addition, the growth of *X. citri* was significantly reduced in leaves of transgenic plants expressing Mthionin compared to that of control plants (Figure 5), which suggests that our Mthionin can kill *X. citri* or inhibit its growth even after bacteria invade the intracellular region bypassing stomata. We have demonstrated earlier that transgenic citrus overexpressing flagellin receptor NbFLS2 cannot inhibit *X. citri* growth or canker development when introduced through infiltration (Hao et al., 2016). Furthermore, with spray inoculation of *X. citri* at 10^6^ CFU/ml, which more closely resembles the natural infection process through stomata, the transgenic plants expressing Mthionin showed very few canker lesions compared to the control plants which developed typical canker symptom. Taken together, our results demonstrated that expression of the Mthionin in transgenic citrus conferred resistance to citrus canker.

HLB challenge studies can be performed by graft transmission or psyllid feeding transmission. Graft inoculation efficiencies generally appear higher compared to psyllid feeding inoculation (Lopes and Frare, 2008; Albrecht et al., 2014). Studies showed that 100% infection was observed 4 months after graft inoculation of 8-month-old Valencia in the greenhouse (Coletta-Filho et al., 2010). Recently it has been demonstrated that Las was detected in roots before leaves, and Las causes root damage prior to development of visible foliar symptoms (Johnson et al., 2014). In our experiment, 6 months after inoculation, Las was detected in some roots (data not shown). Nine months after inoculation, Las was detected in the roots of most plants (Table S1). Graft inoculation failed to confer detectable root Las in one wildtype control plant, despite high Las in rough lemon leaves with a Ct value 24.4 (data not shown), but uneven Las distribution and associated sampling error may be responsible. Similarly some transgenic plants had non-detectable Las, and so further analysis compared only plants with Las detected in roots. Compared to control plants, significantly lower Las titer was detected in roots of transgenic Carrizo rootstocks, suggesting that the Mthionin inhibited Las growth. Twelve months after inoculation, both leaf and root samples were assessed for Las presence. Only one of thirty-four plants showed unsuccessful graft inoculation. The control plants showed higher Las titer in old and young leaves as well as roots compared to transgenic plants expressing Mthionin (Figure 6B). All the transgenic rootstock expressing Mthionin reduced Las titer compared with control plants. Interestingly, some new rough lemon leaves grafted on transgenic rootstock including Thionin-C14-1, 2, 3 5 G1, G5, G6, and H6 have non-detectable Las titer (Table S2), even though the scions were infected prior to grafting and are not transgenic. It is not clear whether thionin can transport to the scion from the transgenic rootstock and reduce Las titer, and this will be elucidated in further studies using antibodies specific to Mthionin.

In summary, the expression of Mthionin appears to provide some protection for citrus plants against two devastating citrus diseases: citrus canker and HLB. We provide clear evidence that transgenic plants expressing Mthionin displayed canker resistance. This transgene is also promising for HLB resistance as low Las titer was detected in the roots of transgenic plants 9 and 12 months after grafting with Las-infected scions. Further evaluations are underway to determine whether HLB resistance can be sustained in the greenhouse and field and whether this effect is commercially significant.

## Author contributions

GH, GG, and ES conceived and designed the experiments. GH performed the experiments. GH analyzed the data. GH and ES contributed reagents/materials/analysis tools. GH and ES wrote the manuscript.

### Conflict of interest statement

The authors declare that the research was conducted in the absence of any commercial or financial relationships that could be construed as a potential conflict of interest.

## Supplementary material

Table S1 **Root Las detection using qPCR and HLB symptom in control plants and transgenic plants expressing Mthionin nine months after graft inoculation**.

Table S2 **Las detection from leaves and roots in control plants and transgenic plants expressing Mthionin twelve months after graft inoculation**.

Figure S1 **Diagram for Mthionin construct in the binary vector pBinARS/Plus**. Designations, LB is left border; Ubi3-p is ubiquitin promoter; NPTII is neomycin phosphotransferase gene (confers kanamycin resistance); Ubi3-t is ubiquitin terminator; D35S-p is double CaMV 35S promoter; Nos-t is nos terminator. RB, right border. Thin arrows indicate position of PCR primers to confirm gene integration located between D35S promoter and Nos terminator regions. Thick arrow heads indicate primers for RT-PCR and RT-qPCR located in Mthionin sequence and nos terminator regions.

Figure S2 **PCR amplification of Mthionin gene**. Total DNA was amplified with primers designed spanning from within the D35S promoter to the 3′ end of the Nos terminator. M, DNA marker; P, Positive control using plasmid as template; W, Water as negative control; N, Non-transformed plant as negative control; Lane 1–15, Transgenic lines containing Mthionin.
