# Supplementary material for: Overexpression of a Modified Plant Thionin Enhances Disease Resistance to Citrus Canker and Huanglongbing (HLB)
Source: Front Plant Sci. 2016 Jul 22;7:1078. doi: 10.3389/fpls.2016.01078 (PMC4956653; doi:10.3389/fpls.2016.01078)
Supplement: Table S1 — Root Las detection using qPCR and HLB symptom in control plants and transgenic plants expressing Mthionin nine months after graft inoculation. [file Table1.docx]

**Table S1 Root Las detection using qPCR and HLB symptom in control plants and transgenic plants expressing Mthionin nine months after graft inoculation**

| Plant | Ct_Las_ | Ct_CD_ | Symptoms^a^ |
| --- | --- | --- | --- |
| Wildtype Control l | 36.6±3.66 | 19.5±0.34 | B |
| Wildtype Control 2 | 25.6±1.61 | 19.9±0.31 | B |
| Wildtype Control 3 | 24.9±2.65 | 20.7±0.33 | B |
| ^b^CKT1-a | 28.5±0.22 | 19.6±1.59 | B, C |
| CKT1-b | 32.4±0.35 | 20.2±0.98 | B |
| CKT1-c | 34.1±0.33 | 20.3±0.57 | B |
| CKT2-a | 28.0±0.27 | 19.5±0.26 | B, C |
| CKT2-b | 27.6±0.09 | 19.1±0.30 | B, C |
| CKT2-c | 23.8±0.07 | 18.5±0.52 | B |
| MThionin-C14-1 | 37.6±1.21 | 19.9±1.95 | B |
| MThionin-C14-2^*^ | 40.0±0.00 | 18.9±0.32 | B, C |
| MThionin-C14-3 | 37.9±1.23 | 19.9±0.24 | B |
| MThionin-C14-4 | 38.3±2.43 | 19.7±1.55 | B |
| MThionin-C14-5 | 36.6±4.88 | 19.6±0.28 | B |
| MThionin-C14-6 | 36.1±0.22 | 20.1±1.88 | B |
| MThionin-C14-7 | 38.5±2.18 | 20.1±0.28 | B |
| MThionin-G1 | 38.7±1.90 | 19.8±0.21 | B, C |
| MThionin-G2 | 34.4±6.40 | 19.2±0.25 | B |
| MThionin-G3 | 35.5±6.42 | 19.8±0.20 | B |
| MThionin-G4 | 39.3±0.94 | 19.4±0.30 | B, |
| MThionin-G5 | 36.0±3.60 | 20.5±0.32 | B |
| MThionin-G6 | 38.7±1.85 | 19.7±0.13 | B, C |
| MThionin-H1 | 39.2±1.13 | 20.2±0.21 | B |
| MThionin-H2 | 38.4±0.89 | 20.0±0.46 | B |
| MThionin-H3 | 38.7±0.12 | 19.3±0.19 | B |
| MThionin-H4 | 32.2±2.02 | 19.3±0.40 | B |
| MThionin-H5 | 34.2±5.74 | 18.9±1.79 | B |
| MThionin-I1 | 34.4±1.38 | 19.4±2.13 | B |
| MThionin-I2^*^ | 40.0±0.00 | 20.0±0.41 | B |
| MThionin-I3 | 36.9±4.45 | 19.9±0.20 | B |
| MThionin-I4 | 39.4±0.81 | 19.1±0.20 | B |
| MThionin-C26-1 | 33.8±0.90 | 19.0±2.33 | B |
| MThionin-C26-2 | 37.0±1.05 | 19.1±2.77 | B |
| MThionin-C26-3^*^ | 40.0±1.05 | 21.8±2.23 | B |

^a^HLB symptom: observed in rough lemon leaves. B represents for blotchy mottle; C represents for chlorosis

^b^CKT1 and CKT2: Transgenic plants expressing vector

The mean threshold cycle values (Ct) with standard deviation from two replicates are listed.

^*^ These plants were not included in the comparisons analysis in Fig. 6A because no detection of Las may result from graft inoculation failure.

**Table S2 Las detection from leaves and roots in control plants and transgenic plants expressing Mthionin twelve months after graft inoculation**

| Plant | Old leaf Ct_Las_ | New leaf Ct_Las_ | Root Ct_Las_ |
| --- | --- | --- | --- |
| Wildtype Control l | 19.5±0.84 | 20.9±0.31 | 24.3±1.31 |
| Wildtype Control 2 | 20.3±1.13 | 21.3±0.61 | 24.0±1.39 |
| Wildtype Control 3 | 17.5±0.43 | 17.7±0.91 | 24.0±1.39 |
| ^b^CKT1-a | 19.4±0.10 | 19.6±0.00 | 26.6±0.72 |
| CKT1-b | 19.9±0.54 | 20.2±0.18 | 27.8±0.30 |
| CKT1-c | 19.2±0.81 | 20.2±0.77 | 25.2±2.40 |
| CKT2-a | 18.8±1.15 | 23.0±0.57 | 23.2±2.72 |
| CKT2-b | 15.3±0.48 | 19.2±1.19 | 22.9±1.63 |
| CKT2-c | 19.5±0.44 | 20.2±0.35 | 26.1±0.35 |
| MThionin-C14-1 | 32.1±0.49 | 40.0±0.00 | 40.0±00 |
| MThionin-C14-2 | 31.7±1.50 | 40.0±00 | 40.0±00 |
| MThionin-C14-3 | 36.8±4.50 | 40.0±00 | 39.1±1.31 |
| MThionin-C14-4 | 31.8±0.65 | 38.3±0.65 | 40.0±00 |
| MThionin-C14-5 | 30.2±0.53 | 38.6±1.97 | 40.0±00 |
| MThionin-C14-6 | 24.0±0.67 | 21.4±1.31 | 40.0±00 |
| MThionin-C14-7 | 38.3±2.34 | 34.7±0.88 | 40.0±00 |
| MThionin-G1 | 33.0±0.90 | 40.0±0.00 | 37.3±3.75 |
| MThionin-G2 | 17.2±0.85 | 23.3±0.69 | 36.3±0.97 |
| MThionin-G3 | 15.5±0.18 | 22.0±0.28 | 38.9±1.49 |
| MThionin-G4 | 19.1±0.54 | 22.5±0.35 | 37.5±0.03 |
| MThionin-G5 | 36.6±4.87 | 38.8±1.76 | 31.9±0.78 |
| MThionin-G6 | 33.1±0.56 | 40.0±0.00 | 34.3±4.78 |
| MThionin-H1 | 22.9±1.00 | 27.3±1.15 | 34.3±1.50 |
| MThionin-H2 | 17.9±0.73 | 24.6±0.07 | 38.9±1.50 |
| MThionin-H3^*^ | 40.0±0.00 | 40.0±0.00 | 40.0±0.00 |
| MThionin-H4 | 21.0±1.93 | 29.0±1.13 | 30.9±1.26 |
| MThionin-H5 | 36.3±5.29 | 40.0±0.00 | 38.9±1.58 |
| MThionin-I1 | 38.8±1.69 | 37.3±3.85 | 38.5±2.07 |
| MThionin-I2 | 35.3±3.51 | 31.3±2.74 | 40.0±0.00 |
| MThionin-I3 | 15.6±0.06 | 20.8±0.10 | 38.5±2.14 |
| MThionin-I4 | 38.8±1.64 | 36.4±1.46 | 40.0±0.00 |
| MThionin-C26-1 | 18.0±1.12 | 26.0±0.13 | 28.4±1.26 |
| MThionin-C26-2 | 31.8±1.16 | 31.9±0.77 | 40.0±0.00 |
| MThionin-C26-3 | 36.6±0.71 | 37.4±0.24 | 40.0±0.00 |

^b^CKT1 and CKT2: Transgenic plants expressing vector

^*^The plant was not included in the comparisons analysis in Fig. 6B because of graft inoculation failure

The mean threshold cycle values (Ct) with standard deviation from two replicates are listed.
