# Supplementary figures and images for: Overexpression of a Modified Plant Thionin Enhances Disease Resistance to Citrus Canker and Huanglongbing (HLB)
Source: Front Plant Sci. 2016 Jul 22;7:1078. doi: 10.3389/fpls.2016.01078 (PMC4956653; doi:10.3389/fpls.2016.01078)

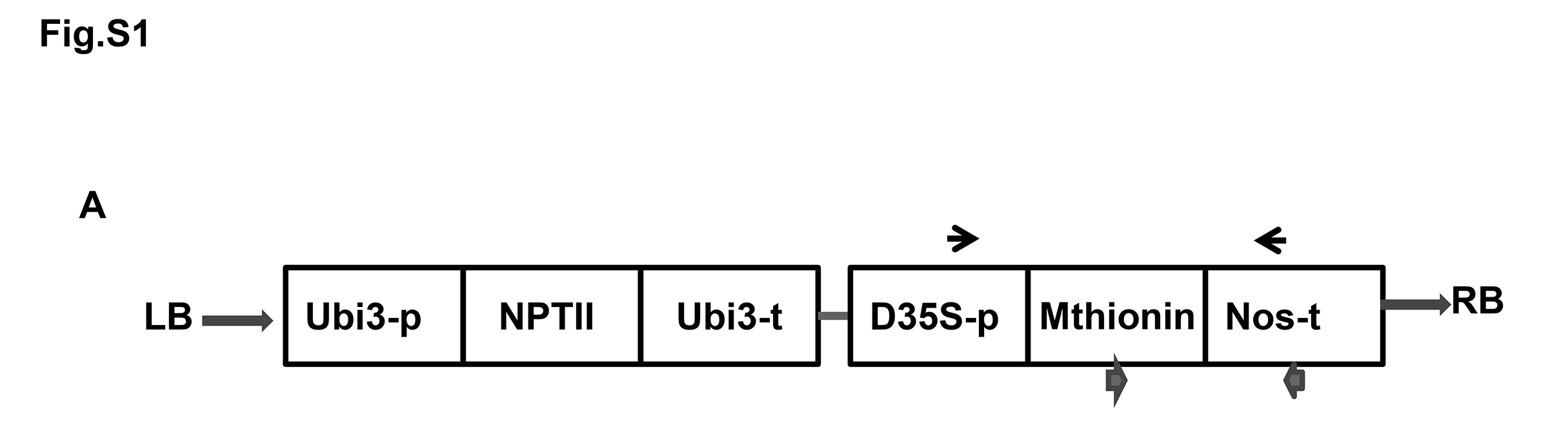

Supplement: Figure S1 — Diagram for Mthionin construct in the binary vector pBinARS/Plus. Designations, LB is left border; Ubi3-p is ubiquitin promoter; NPTII is neomycin phosphotransferase gene (confers kanamycin resistance); Ubi3-t is ubiquitin terminator; D35S-p is double CaMV 35S promoter; Nos-t is nos terminator. RB, right border. Thin arrows indicate position of PCR primers to confirm gene integration located between D35S promoter and Nos terminator regions. Thick arrow heads indicate primers for RT-PCR and RT-qPCR located in Mthionin sequence and nos terminator regions. [file Image1.tif]

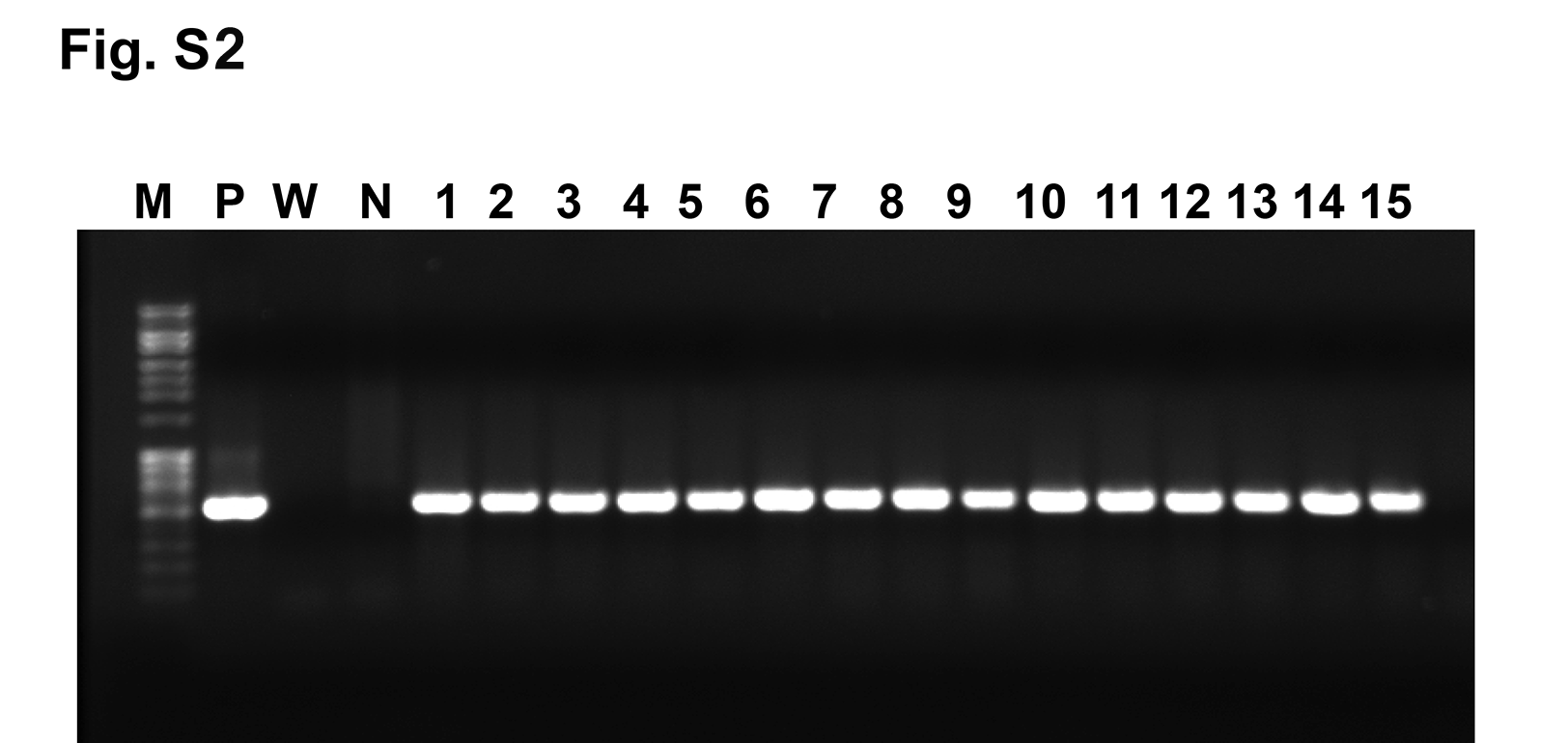

Supplement: Figure S2 — PCR amplification of Mthionin gene. Total DNA was amplified with primers designed spanning from within the D35S promoter to the 3′ end of the Nos terminator. M, DNA marker; P, Positive control using plasmid as template; W, Water as negative control; N, Non-transformed plant as negative control; Lane 1–15, Transgenic lines containing Mthionin. [file Image2.tif]
